# Individualized Analysis of Nipple‐Sparing Mastectomy Versus Modified Radical Mastectomy Using Deep Learning

**DOI:** 10.1002/cai2.70002

**Published:** 2025-03-26

**Authors:** Enzhao Zhu, Linmei Zhang, Pu Ai, Jiayi Wang, Chunyu Hu, Huiqing Pan, Weizhong Shi, Ziqin Xu, Yidan Fang, Zisheng Ai

**Affiliations:** ^1^ School of Medicine Tongji University Shanghai China; ^2^ Shanghai Engineering Research Center of Tooth Restoration and Regeneration, Tongji Research Institute of Stomatology, Department of Prosthodontics, Shanghai Tongji Stomatological Hospital, Dental School Tongji University Shanghai China; ^3^ Tenth People's Hospital of Tongji University, School of Medicine Tongji University Shanghai China; ^4^ Shanghai Hospital Development Center Shanghai China; ^5^ Columbia University New York USA; ^6^ University of Warwick Coventry UK; ^7^ Department of Medical Statistics, School of Medicine Tongji University Shanghai China

**Keywords:** breast cancer, deep learning, modified radical mastectomy, neoadjuvant systemic treatment, nipple‐sparing mastectomy

## Abstract

**Background:**

This study aimed to evaluate the impact of nipple‐sparing mastectomy (NSM) and modified radical mastectomy (MRM) on individual survival outcomes and to assess the potential of neoadjuvant systemic therapy (NST) in reducing surgical intervention requirements.

**Methods:**

To develop treatment recommendations for breast cancer patients, five machine learning models were trained. To mitigate bias in treatment allocation, advanced statistical methods, including propensity score matching (PSM) and inverse probability treatment weighting (IPTW), were applied.

**Results:**

NSM demonstrated either superior or noninferior survival outcomes compared with MRM across all breast cancer stages, irrespective of adjustments for IPTW and PSM. Among all models and National Comprehensive Cancer Network guidelines, the Balanced Individual and Mixture Effect (BIME) for survival regression model proposed in this study showed the strongest protective effects in treatment recommendations, as evidenced by an IPTW hazard ratio of 0.39 (95% CI: 0.26–0.59), an IPTW risk difference of 19.66% (95% CI: 18.20–21.13), and an IPTW difference in restricted mean survival time of 17.77 months (95% CI: 16.37–19.21). NST independently reduced the probability of surgical intervention by 1.4% (95% CI: 0.9%–2.0%), with the greatest impact observed in patients with locally advanced breast cancer, in whom a 4.5% reduction (95% CI: 3.8%–5.2%) in surgical selection was noted.

**Conclusions:**

The BIME model provides superior accuracy in recommending surgical approaches for breast cancer patients, leading to improved survival outcomes. These findings underscore the potential of BIME to enhance clinical decision‐making. However, further investigation incorporating comprehensive prognostic evaluation is needed to optimize the surgical selection process and refine its clinical utility.

## Graphical abstract

This study aims to explore the overall survival (OS) and breast cancer‐specific survival (BCSS) advantages of nipple‐sparing mastectomy (NSM) and modified radical mastectomy (MRM) at the individual level and to quantify the extent to which neoadjuvant systemic treatment (NST) can be used for surgical downgrading. Five machine learning models were trained to make treatment recommendations for patients with breast cancer. The Balanced Individual and Mixture Effect for survival regression (BIME) demonstrated the strongest and statistically significant protective effect. NST independently resulted in a 1.4% (95% CI, 0.9%–2.0%) surgical downgrade effect and had the most pronounced effect on the surgical choice of locally advanced breast cancer patients. Our study demonstrated that BIME could make better surgery recommendations for breast cancer patients and can extend the survival time, which can be used to assist in clinical decision‐making.

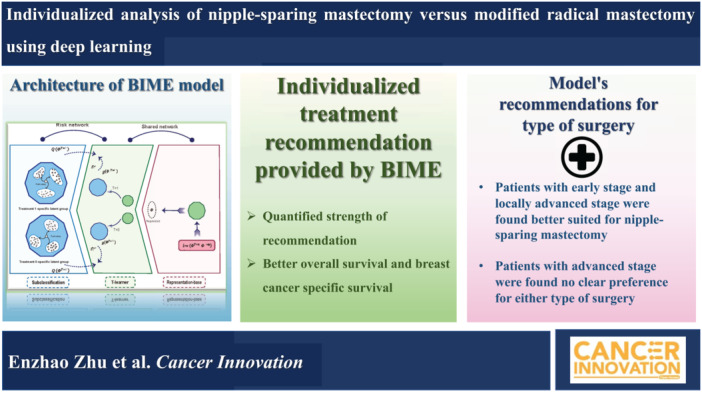

**Abbreviations**
BCSM: breast cancer‐specific mortality
BCSS: breast cancer‐specific survival
BIME: Balanced Individual and Mixture Effect for Survival Regression
BITES: Balanced Individual Treatment Effect for Survival Data
CATE: conditional average treatment effect
Consis: those who received the recommended treatment
CPH: cox proportional hazards model
DCM: deep cox mixture model
DL: deep learning
dRMST: difference in restricted mean survival time
EBC: early breast cancer
ER: estrogen receptor
HER2: human epidermal growth factor receptor 2
IBS: integrated Brier score
Inconsis: those who received a non‐recommended treatment
IPM: Integral Probability Metrics
IPTW: inverse probability treatment weighting
IQR: interquartile range
ITE: individual treatment effect
K‐M: Kaplan–Meier
LABC: locally advanced breast cancer
MBC: metastatic breast cancer
MCEM: Monte Carlo expectation‐maximization
MRM: modified radical mastectomy
NCCN: National Comprehensive Cancer Network
NDE: natural direct effect
NSM: nipple‐sparing mastectomy
NST: neoadjuvant systemic therapy
OS: overall survival
PHA: proportional hazards assumption
PR: progesterone receptor
PSM: propensity score matching
RCT: randomized‐controlled trial
RD: risk difference
RSF: random survival forest
SD: standard deviation
SEER: Surveillance, Epidemiology, and End Result
SMD: standardized mean difference
STROBE: Strengthening the Reporting of Observational Studies in Epidemiology
TaR: time at risk
T‐learner: two‐learner

## 1 Introduction

Breast cancer remains the most frequently diagnosed malignancy impacting women's health globally [1]. Subcutaneous mastectomy, initially introduced as a treatment for benign breast conditions, has undergone significant evolution and is now widely utilized in women with a marked familial predisposition to breast cancer [2]. In 1980, Gentil et al. introduced the concept of nipple‐sparing mastectomy (NSM) for both prophylactic and therapeutic applications [3], emphasizing its potential for superior cosmetic results [4].

Although NSM offers notable cosmetic advantages, concerns persist regarding its efficacy and oncological safety [5]. The National Comprehensive Cancer Network (NCCN) endorses NSM primarily for preventive indications and for cases of early breast cancer (EBC) with peripheral tumor localization and favorable prognosis [6]. A large‐scale retrospective analysis using propensity score matching suggested improved overall survival (OS) with NSM compared with modified radical mastectomy (MRM) in patients with locally advanced breast cancer (LABC), although the difference did not reach statistical significance [7]. Furthermore, recurrence rates—both locoregional and distant—remain acceptably low following NSM [8, 9], reinforcing its oncological safety profile and its association with favorable OS and breast cancer‐specific survival (BCSS) outcomes [10]. A meta‐analysis of 1202 participants, controlling for age and tumor stage, revealed a trend favoring NSM over MRM (hazard ratio [HR]: 0.71, 95% confidence interval [CI]: 0.46–1.13) [5]. Recent data from the American College of Surgeons and the American Cancer Society highlight an increasing preference for NSM, particularly in patients receiving neoadjuvant systemic treatment (NST) [11], underscoring the growing interest in the feasibility of NSM post‐NST [12].

The absence of randomized‐controlled trials (RCTs) directly comparing NSM with MRM limits the availability of definitive evidence regarding the feasibility of NSM and the potential for NST to minimize the extent of required surgical intervention. In the absence of such trials, observational data offer a viable alternative for hypothesizing individualized treatment outcomes, addressing the ethical and financial barriers to conducting RCTs [13, 14]. Meta‐learners, notably the two‐learner (T‐learner) approach, are widely used to estimate individual treatment effects (ITEs) under conditions of minimal patient heterogeneity, thereby approximating a quasi‐RCT framework to infer the conditional average treatment effect (CATE). Advances in deep learning (DL) have introduced representation‐based balancing methods designed to mitigate confounding effects, enabling more accurate and unbiased evaluations of treatment effects [15, 16, 17].

This study applies advanced DL models to investigate the individualized benefits of NSM compared with MRM, focusing on OS and BCSS. Additionally, it examines the potential of NST to enable less invasive surgical strategies, providing novel insights into personalized surgical approaches for breast cancer management.

## 2 Methods and Materials

### 2.1 Study Design and Data Source

This study utilized data from the Surveillance, Epidemiology, and End Result (SEER) database, which compiles comprehensive cancer patient information from 18 regions across the United States [18]. Reporting adhered to the standards of the Strengthening the Reporting of Observational Studies in Epidemiology (STROBE) initiative [19]. Using a population‐based sample, the analysis incorporated DL techniques to evaluate the ITE of NSM versus MRM in breast cancer patients. The study population consisted of female patients diagnosed with a single primary breast cancer—ductal, lobular, or mixed carcinoma—between 2010 and 2017. All participants underwent either NSM or MRM. The studies involving human participants were approved by the National Cancer Institution (approval ID: SAR0059979). The studies were conducted in accordance with the local legislation and institutional requirements. Written informed consent for participation was not needed for this study in accordance with national legislation and institutional requirements.

**Figure 1:**
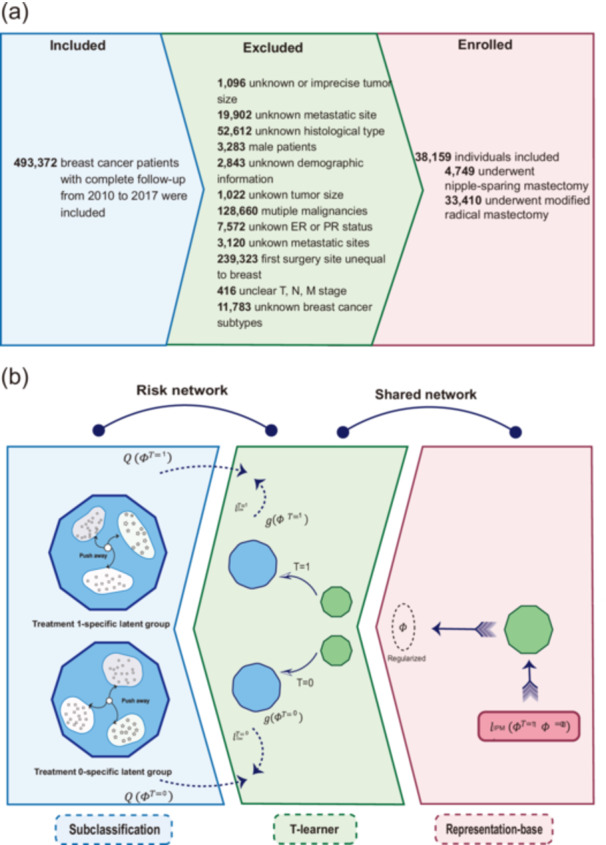
Diagram of the inclusion procedure and model architecture. (a) Inclusion process. (b) Architecture of Balanced Individual and Mixture Effect for survival regression. ER, estrogen receptor; PR, progesterone receptor.

Several exclusion criteria were applied to ensure data quality and relevance. Individuals with missing demographic information, unknown receptor statuses (including human epidermal growth factor receptor 2 [HER2], estrogen receptor [ER], or progesterone receptor [PR]), and those with carcinoma in situ or bilateral breast cancer were excluded. Additionally, exclusions encompassed patients with unclear TNM staging, undetermined tumor size, or unknown metastasis or metastatic sites. Participants were also excluded if axillary lymph node status was undefined, systemic treatment or radiotherapy data were unavailable, follow‐up data were incomplete, or if multiple malignancies were present (Figure 1a).

Cancer staging adhered to the 7th edition of the American Joint Committee on Cancer Staging Manual. Survival outcomes were analyzed using OS and BCSS. Patients were censored as of December 31, 2019. Given the completeness of the data set, imputation methods were not required.

LABC was defined as stage IIB (T3N0) through stages IIIA, IIIB, and IIIC, characterized by larger tumors and/or extensive nodal involvement. In contrast, early breast cancer was classified as in situ, Stage I, or Stage IIA [20].

### 2.2 Deep Learning Algorithms and Related Works

The T‐learner uses two independently trained models—statistical or machine learning frameworks—to estimate conditional outcomes for different treatment arms. Each model is trained separately on distinct intervention groups [21]. The CATE is calculated as shown in Equation 1:

(1) $$\mathrm{CATE}\left(x\right)=m_{1}\left(x\right)-m_{0}(x)$$

In Equation 1, $m_{1}\left(x\right)$ and $m_{0}\left(x\right)$ correspond to the models trained on the MRM and NSM groups, respectively, and x represents the baseline characteristics of an individual patient.

The performance of the T‐learner is highly dependent on the accuracy of the base models [13]. Notably, when there is significant imbalance between treatment groups, the model trained on the smaller group often underperforms. Furthermore, although the T‐learner addresses confounding factors, it may still fail to balance generative distributions affected by biased treatment allocation. Prior studies have demonstrated that balancing generative distributions across treatment groups improves both covariate representations [22] and latent features in predictive models [23]. To address these challenges, the Balanced Individual Treatment Effect for Survival data (BITES) approach [24] incorporates Integral Probability Metrics (IPM). However, BITES assumes a proportional hazards assumption (PHA), which can be restrictive. Alternatively, the Deep Cox Mixture model (DCM) [25] leverages multiple Cox regressions combined through a gating function to assign individual *i* to specific latent clusters. By encoding covariates via a neural network, DCM learns latent representations and computes log hazard ratios using weighted components of the mixture. Monte Carlo expectation‐maximization (MCEM) is used to sample parameters, allowing DCM to replace the PHA with a conditional proportional hazards assumption that applies within each latent group.

In this study, we propose a new architecture, the Balanced Individual and Mixture Effect (BIME), to advance causal inference methods. The architecture, presented in Figure 1b, consists of a shared central network and two specialized risk networks. The central network uses IPM to optimize the p‐Wasserstein distance between treatment arms, achieving balanced generative distributions. The loss function is computed using a smoothed optimal transport approach. Balanced latent features are then input into two multilayer perceptron networks, which serve as the risk networks, to predict the CATE within a T‐learner framework. To capture patient‐specific hazard profiles, BIME forms minibatches of posterior distributions, using a mixture of K neural networks to model diverse risk factors. The Q(·)function [25] is applied to refine patient representations, while MCEM optimizes the mixture weights and updates model parameters. This approach minimizes heterogeneity within each latent group by ensuring similar log hazard ratios for patients in the same cluster [13]. Subsequently, the negative Cox partial log‐likelihood is computed separately for each treatment group. The final BIME loss function integrates four key components as in Equation 2:

(2) $$\begin{array}{lll} l_{\text{BIME}}\left(x_{i},y_{i},E_{i},T_{i}\right) & = & ql_{\text{Cox}\;}^{T=0}\left(h_{0}(\Phi (x)),Y^{T=0},E^{T=0}\right)\\ & & +\;\left(1-q\right)l_{\text{Cox}}^{T=1}\left(h_{1}\left(\Phi \left(x\right)\right),Y^{T=1},E^{T=1}\right)\\ & & +\;\alpha l_{{\text{IPM}}_{\epsilon }^{p}}\left(\Phi^{T=1},\Phi^{T=0}\right)\\ & & +\;Q^{T=0}\left(\Phi^{T=0}\right)\\ & & +\;Q^{T=1}\left(\Phi^{T=1}\right) \end{array}$$

In Equation 2, the failure event is denoted as E, the observed survival times are represented by y, the division of individuals within the control cohort (T=0) is denoted by q, and the negative Cox partial log‐likelihood is represented by $l_{\text{Cox}}$. The regularization quality of the IPM is controlled by the parameter α, while the hazard function h(·) represents the regularized latent representation Φ.

During the inference phase, BIME computes treatment‐specific baseline hazards and predicts outcomes for two treatment options: MRM and NSM. The primary outcome, termed time at risk (TaR), reflects the duration required for a patient to reach 90% mortality. The ITE is calculated as the difference in TaR between MRM and NSM for each patient. Specifically, the ITE for patient *i* is given by Equation 3:

(3) $${\mathrm{ITE}}_{i}={\mathrm{TaR}}_{i}^{\mathrm{MRM}}-{\mathrm{TaR}}_{i}^{\mathrm{NSM}}$$

In Equation (3), ${\mathrm{TaR}}_{i}^{\mathrm{MRM}}$ represents the time at risk under MRM and ${\mathrm{TaR}}_{i}^{\mathrm{NSM}}$ denotes the time at risk under NSM.

### 2.3 Treatment Recommendation Models

The patient cohort was randomly divided into two subsets: a training set comprising 70% of the data and a testing set comprising the remaining 30%. Model training used fivefold cross‐validation to enhance robustness. Specifically, the training data set was partitioned into five subsets, with four subsets used for training and the remaining subset reserved for validation in each iteration. This process was repeated five times, ensuring that each subset served as the validation set exactly once. To mitigate the risk of overfitting and enhance generalizability, early stopping was implemented. Training was halted if validation loss did not improve after 1000 iterations. This strategy ensured computational efficiency and optimized the model's performance on unseen data.

We trained several models, including BIME, BITES, DeepSurv [26], the Cox proportional hazards model (CPH), and the random survival forest (RSF). Training and inference for DeepSurv, CPH, and RSF were harmonized with the T‐learner framework to ensure uniformity in model evaluation. The calculation method for the ITE was standardized across all models. To assess the outcomes of treatment recommendations, patients were stratified into two groups based on concordance with model recommendations: those who received the recommended treatment (Consis) and those who received a non‐recommended treatment (Inconsis). This categorization facilitated a systematic evaluation of treatment efficacy relative to model‐guided recommendations.

### 2.4 Statistical Analysis

Statistical analyses were conducted using R software version 4.13 and Python version 3.8. For continuous variables, data distributions determined whether results were presented as medians with interquartile ranges (IQRs) or means with standard deviations (SDs). Categorical variables were expressed as counts and percentages (%). To mitigate treatment selection bias and address potential confounding, inverse probability of treatment weighting (IPTW) and propensity score matching (PSM) were used. These approaches ensured balance between treatment groups, facilitating adjustments for confounders. Logistic regression models were used to estimate propensity scores, incorporating variables known to influence prognosis [27] or clinical treatment decisions [20], such as age, breast cancer subtype, tumor grade, axillary lymph node status, and TNM stage [1, 28]. Adjustment for confounders was critical for two reasons. First, imbalance in baseline characteristics arising from selection bias could skew survival outcomes between treatment groups, resulting in biased effect estimates [29]. Second, such imbalances could influence the evaluation of treatment recommendation performance, as one group (e.g., the Consis group) might inherently show more favorable prognostic factors. Thus, standardization of survival expectations across groups was necessary to derive valid causal inferences. IPTW incorporated propensity scores as weights within outcome models, while PSM used 1:1 nearest‐neighbor matching based on propensity score similarity. Kaplan–Meier (K–M) survival curves were compared using the log‐rank test and the Anderson–Darling test was utilized to assess data normality in large samples [30]. Statistical significance was defined as a two‐tailed *p* < 0.05. Treatment recommendation models were developed using Python libraries, including PyTorch and scikit‐survival. Statistical analyses were performed using R packages, including survival, ipw, survRM2, survminer, PSweight, and MatchIt.

## 3 Results

### 3.1 Demographic Characteristics and Clinicopathological Features

The study cohort comprised 38,159 patients diagnosed with breast cancer, with a median follow‐up duration of 59 months (IQR: 36–87 months). Over the follow‐up period, the overall mortality rate was 21.8% (95% CI: 21.3%–22.2%), while the breast cancer‐specific mortality (BCSM) rate was 14.9% (95% CI: 14.5%–15.3%). Table 1 provides a comprehensive summary of the baseline clinical characteristics of all patients, highlighting those who underwent NSM or MRM. Regarding surgical approaches, 4749 patients (12.4%) received NSM and 33,410 patients (87.6%) underwent MRM. Notably, the NSM cohort showed significantly lower overall mortality (NSM vs. MRM: 4.0% vs. 24.3%, *p* < 0.0001) and BCSM (NSM vs. MRM: 3.2% vs. 16.6%, *p* < 0.0001) compared with the MRM cohort.

**Table 1:** Baseline demographic and pathological features.

Features	Nipple‐sparing mastectomy (*n* = 4749)	Modified radical mastectomy (*n* = 33,410)	Statistics	*p*
Age, mean (SD), y	49.4 (10.7)	57.4 (14.1)	−46.1^a^	< 0.001***
Tumor size, mean (SD), mm	23.4 (20.0)	36.8 (29.1)	−40.5^a^	< 0.001***
Married, *n* (%)	3273 (68.9)	18,086 (54.1)	368.3^b^	< 0.001***
Race–White, *n* (%)	3744 (78.8)	25,278 (75.7)	22.8^b^	< 0.001***
Income–Higher than 70,000$, *n* (%)	2245 (47.3)	9968 (29.8)	580.2^b^	< 0.001***
Grade, *n* (%)			427.5^b^	< 0.001***
I	921 (19.4)	3538 (10.6)		
II	2154 (45.4)	13,990 (41.9)		
III	1669 (35.1)	15,765 (47.2)		
IV	5 (0.1)	117 (0.4)		
Location, *n* (%)			197.3^b^	< 0.001***
Upper outer quadrant	1607 (33.8)	10,725 (32.1)		
Upper inner quadrant	645 (13.6)	2912 (8.7)		
Lower outer quadrant	413 (8.7)	2385 (7.1)		
Lower inner quadrant	231 (4.9)	1349 (4.0)		
Central/overlapping	1124 (23.7)	9810 (29.4)		
Nipple/axillary tail	20 (0.4)	254 (0.8)		
Other/unknown	709 (14.9)	5975 (17.9)		
T stage, *n* (%)			1673^b^	< 0.001***
T1	2677 (56.4)	9591 (28.7)		
T2	1677 (35.3)	15,057 (45.1)		
T3	350 (7.4)	5583 (16.7)		
T4	45 (0.9)	3171 (9.5)		
N stage, *n* (%)			3719.2^b^	< 0.001***
N0	3216 (67.7)	8394 (25.1)		
N1	1229 (25.9)	14,425 (43.2)		
N2	219 (4.6)	6384 (19.1)		
N3	84 (1.8)	4197 (12.6)		
M stage, *n* (%)			161.0^b^	< 0.001***
M0	4720 (99.4)	31,906 (95.5)		
M1	29 (0.6)	1496 (4.5)		
TNM stage, *n* (%)			3943.5^b^	< 0.001***
IA	2145 (45.2)	4417 (13.2)		
IB	130 (2.7)	542 (1.6)		
IIA	1274 (26.8)	6247 (18.7)		
IIB	701 (14.8)	7561 (22.6)		
IIIA	357 (7.5)	7431 (22.2)		
IIIB	34 (0.7)	2045 (6.1)		
IIIC	79 (1.7)	3669 (11.0)		
IV	29 (0.6)	1498 (4.5)		
Distant metastasis, *n* (%)			…^d^	0.206
Bone	14 (0.3)	851 (2.5)		
Brain	2 (0.0)	47 (0.1)		
Liver	9 (0.2)	280 (0.8)		
Lung	7 (0.1)	343 (1.0)		
Axillary lymph node‐positive, *n* (%)	1455 (30.6)	24,278 (72.7)	3342.7^b^	< 0.001***
ER status–positive, *n* (%)	3952 (83.2)	25,871 (77.4)	81.0^b^	< 0.001***
PR status–positive, *n* (%)	3510 (73.9)	21,793 (65.2)	139.8^b^	< 0.001***
HER status‐positive, *n* (%)	873 (18.4)	7279 (21.8)	28.4^b^	< 0.001***
Neoadjuvant systemic treatment, *n* (%)	1171 (24.7)	9106 (27.3)	14.1^b^	< 0.001***
Follow‐up, median [IQR], month	48 [33, 65]	61 [36, 90]	61,794,385^c^	< 0.001***
Breast cancer‐specific mortality, *n* (%)	150 (3.2)	5535 (16.6)	588.5^b^	< 0.001***
Overall mortality, *n* (%)	191 (4.0)	8110 (24.3)	1000.7^b^	< 0.001***

Abbreviations: a, Welch *t*‐test; b, *χ* ^2^ test with continuity correction; c, Wilcoxon test; d, Fisher's exact test; IQR, interquartile range; SD, standard deviation.

*** *p* < 0.001.

### 3.2 Comparative Outcomes of NSM and MRM Across Subgroups

**Figure 2:**
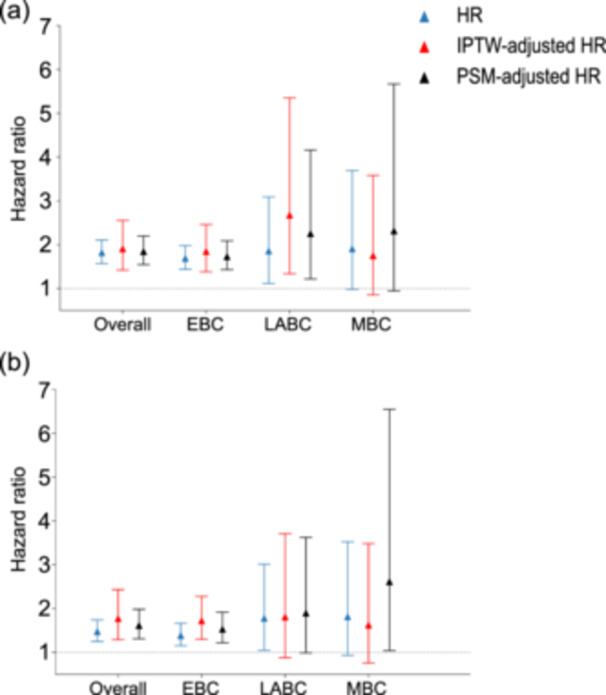
Average treatment effect and treatment heterogeneity. (a) Hazard ratio of overall survival. (b) Hazard ratio of breast cancer‐specific survival. EBC, early breast cancer; HR, multivariate hazard ratio; IPTW, inverse probability treatment weighting; LABC, locally advanced breast cancer; MBC, metastatic breast cancer; PSM, propensity score matching.

The comparative efficacy of NSM and MRM was evaluated across three breast cancer stages: EBC, LABC, and metastatic breast cancer (MBC). OS outcomes are presented in Figure 2a, while BCSS results are presented in Figure 2b. The standardized mean differences (SMDs) before and after IPTW and PSM adjustments are illustrated in Supporting Information S1: Figures S1a and S1b, respectively. After statistical adjustments, covariate balance was achieved (SMD < 0.1 for most variables).

Patients undergoing NSM demonstrated superior OS and BCSS compared with those undergoing MRM across the entire cohort (IPTW HR for OS: 1.90, 95% CI: 1.42–2.55; PSM HR for OS: 1.84, 95% CI: 1.54–2.20; IPTW HR for BCSS: 1.77, 95% CI: 1.29–2.43; PSM HR for BCSS: 1.61, 95% CI: 1.31–1.98). These advantages were evident in the EBC subgroup (IPTW HR for OS: 1.84, 95% CI: 1.38–2.46; PSM HR for OS: 1.73, 95% CI: 1.43–2.09; IPTW HR for BCSS: 1.72, 95% CI: 1.30–2.28; PSM HR for BCSS: 1.53, 95% CI: 1.22–1.92). In the LABC subgroup, NSM was associated with improved OS (IPTW HR for OS: 2.67, 95% CI: 1.33–5.35; PSM HR for OS: 2.25, 95% CI: 1.22–4.16), although no significant differences in BCSS were observed (IPTW HR for BCSS: 1.81, 95% CI: 0.88–3.71; PSM HR for BCSS: 1.89, 95% CI: 0.99–3.52). Similarly, no significant differences were identified for OS (IPTW‐adjusted HR: 1.75, 95% CI: 0.85–3.58) or BCSS (IPTW‐adjusted HR: 1.62, 95% CI: 0.76–3.48) in the MBC subgroup.

### 3.3 Performance of Treatment Recommendation Models

To assess the effectiveness of the treatment recommendation models, several performance metrics were computed, including the HR, difference in restricted mean survival time (dRMST), and risk difference (RD). To address potential confounding factors and ensure balance between the Consis. and Inconsis. groups, IPTW was used. This approach enabled comprehensive covariate adjustment. The primary endpoint was OS assessed over a 10‐year follow‐up period. A summary of model performance can be found in Table 2.

**Table 2:** Detailed model performance and treatment recommendation effects.

Model	IBS^a^	IBS^b^	HR	HR^c^	RD (%)	RD^c^ (%)	dRMST (month)	dRMST^c^ (month)
BIME	**0.05 (0.03–0.07)**	0.14 (0.14–0.15)	**0.64 (0.52–0.79)**	**0.39 (0.26–0.59)**	23.10 (19.20–27.00)	**19.66 (18.20–21.13)**	17.73 (16.25–19.21)	17.77 (16.37–19.21)
BITES	0.05 (0.4–0.08)	0.14 (0.13–0.14)	1.04 (0.95–1.13)	1.05 (0.96–1.15)	8.80 (5.90–11.70)	16.96 (15.50–18.39)	16.38 (15.01–17.76)	16.44 (15.31–17.72)
DeepSurv	0.07 (0.05–0.09)	0.34 (0.34–0.35)	0.98 (0.82–1.08)	0.94 (0.82–1.08)	0.10 (−0.73–0.75)	10.30 (8.49–12.10)	5.09 (2.84–7.33)	**19.07 (17.33–20.71)**
CPH	0.05 (0.04–0.06)	0.11 (0.11–0.12)	1.04 (0.90–1.20)	0.90 (0.65–1.23)	19.30 (16.40–22.20)	14.07 (12.61–15.52)	14.07 (12.61–15.52)	14.21 (12.67–15.55)
RSF	0.05 (0.03–0.06)	**0.11 (0.11–0.12)**	0.80 (0.70–0.91)	0.81 (0.69–0.94)	**24.50 (21.60–27.40)**	17.70 (16.30–19.10)	**17.91 (16.57–19.24)**	17.95 (16.76–19.43)
NCCN	—	—	0.99 (0.86–1.14)	0.68 (0.55–0.84)	9.57 (7.98–11.20)	8.23 (6.71–9.75)	11.89 (10.31–13.48)	14.31 (11.98–16.17)

*Note:* All metrics are calculated based on a 10‐year time horizon. Metrics where the model performs best are indicated in bold. According to NCCN Guidelines Version 4.2023, NSM is recommended for patients with any of the following factors: Grade I and II tumors, human epidermal growth factor receptor‐2‐negative status, N0, or axillary lymph node‐negative status.

Abbreviations: BIME, Balanced Individual and Mixture Effect survival regression; BITES, Balanced Individual Treatment Effect for Survival data; CPH, Cox proportional hazards model; dRMST^c^, difference in restricted survival time, adjusted for all covariates using inverse probability treatment weighting; HR, multivariate hazards ratio; IBS^a^, integrated Brier score for the nipple‐sparing mastectomy treatment group; IBS^b^, integrated Brier score for the modified radical mastectomy treatment group; NCCN, National Comprehensive Cancer Network; RD, risk difference; RSF, random survival forest.

Among the evaluated models, the BIME achieved the lowest integrated Brier score (IBS) within the NSM group (0.05, 95% CI: 0.03–0.07). Conversely, the RSF model showed superior performance within the MRM group, with an IBS of 0.11 (95% CI: 0.11–0.12). Regarding treatment recommendations, BIME suggested NSM for 10,539 patients (92.1%), of whom 9561 patients (83.5%) were classified in the Inconsis. group. By comparison, the following proportions of patients were recommended for NSM by alternative models: BITES for 8657 (75.6%), DeepSurv for 11,014 (96.2%), CPH for 8,753 (76.5%), and RSF for 7,631 (66.7%). Notably, BIME achieved the most favorable results across HR, IPTW‐adjusted HR (HR^c^), and IPTW‐adjusted RD (RD^c^) (HR: 0.64, 95% CI: 0.52–0.79; HR^c^: 0.39, 95% CI: 0.26–0.59; RD^c^: 19.66, 95% CI: 18.20–21.13), while RSF and DeepSurv excelled in dRMST (dRMST of RSF: 17.91, 95% CI: 16.57–19.24) and IPTW‐adjusted dRMST (dRMST^c^) (dRMST^c^ of DeepSurv: 19.07, 95% CI: 17.33–20.71), respectively.

BIME was identified as the most effective model in terms of recommendation performance. This conclusion is supported by its dRMST (BIME's dRMST: 17.73, 95% CI: 16.25–19.21) and dRMSTc (BIME's dRMSTc: 17.77, 95% CI: 16.37–19.21), which were comparable to those of the highest‐performing models in specific subdomains. Furthermore, following adjustment for selection bias, BIME demonstrated superior HR and RD values relative to all other models.

Additionally, we performed a comparison with the latest NCCN guidelines (version 4.2023) [31], evaluating outcomes for patients whose treatments aligned with NCCN recommendations versus those whose treatments deviated. While adherence to NCCN guidelines conferred some protective effects (HR: 0.99, 95% CI: 0.86–1.14; HR^c^: 0.68, 95% CI: 0.55–0.84; RD: 9.57, 95% CI: 7.98–11.20; RD^c^: 8.23, 95% CI: 6.71–9.75; dRMST: 11.89, 95% CI: 10.31–13.48; dRMST^c^: 14.31, 95% CI: 11.98–16.17), these effects were notably inferior to those observed with BIME.

We further examined whether the model's protective effect on patients was modulated by an imbalance in treatment allocation between the two groups. To address this, we delineated the causal pathway underlying the protective effect of BIME in Supporting Information S1: Figure S2. In this analysis, the administered treatments, NSM and MRM, were incorporated as mediator variables, while all other covariates were treated as potential confounders. To assess the impact of BIME's recommendations, we estimated both the natural direct effect (NDE) and the natural indirect effect. Effect sizes were quantified using linear regression, with the corresponding slopes of the regression models presented as measures of effect magnitude. After accounting for the mediating effects of treatment allocation, the protective effect of BIME remained statistically significant. Specifically, the NDE was calculated to be −0.18 (95% CI: −0.18–−0.17), demonstrating that BIME conferred a beneficial outcome independent of the treatments administered.

### 3.4 Causal Inference of NST on Surgical Downgrading

**Figure 3:**
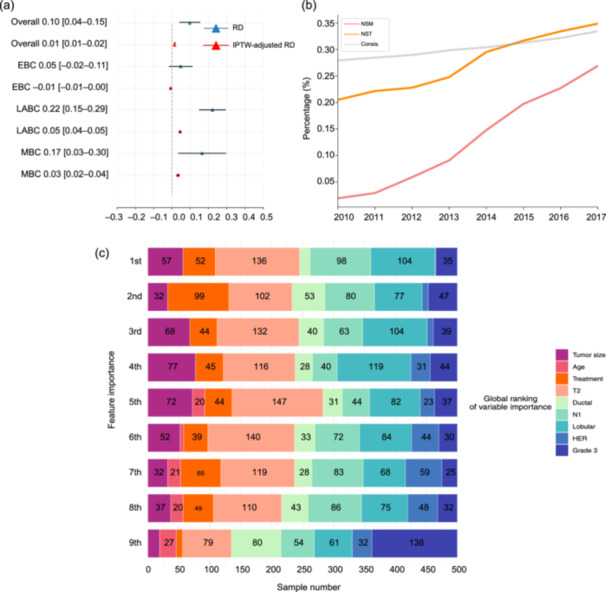
Causal inference of NST to surgical downgrading and model interpretation using SurvSHAP(t). (a) Surgical downgrading effect of NST. (b) Proportion of NSM, Consis., and NST from 2010 to 2017. (c) Model interpretation based on SurvSHAP(t). EBC, early breast cancer; HER, human epidermal growth factor receptor; IPTW, inverse probability treatment weighting; LABC, locally advanced breast cancer; MBC, metastatic breast cancer; NSM, nipple‐sparing mastectomy; NST, neoadjuvant systemic treatment; PSM, propensity score matching and RD, risk difference (probability of being recommended for NSM with NST minus without NST).

Patients were stratified into two groups based on the recommendations provided by the BIME system: those advised to undergo NSM and those recommended for MRM. NST was treated as the primary intervention variable influencing the model's treatment recommendations, enabling the framing of NST's role in surgical downgrading as a binary causal inference problem grounded in the recommendation patterns of BIME. The RD quantified the probability of a patient being recommended for NSM after receiving NST minus the probability of such a recommendation without NST. To isolate the independent effect of NST, the IPTW‐adjusted RD was calculated. The results of these analyses are presented in Figure 3a.

Overall, NST demonstrated a surgical downgrading effect of 9.8% (95% CI: 4.1%–15.5%), with an IPTW‐adjusted effect of 1.4% (95% CI: 0.9%–2.0%). Subgroup analyses revealed no statistically significant effect of NST in patients with EBC (RD: 4.8%, 95% CI: −1.7%–11.3%; IPTW‐adjusted RD: −0.6%, 95% CI: −1.3%–0.1%). However, NST had a more pronounced effect on the surgical decision‐making for patients with LABC (RD: 22.2%, 95% CI: 14.9%–29.5%; IPTW‐adjusted RD: 4.5%, 95% CI: 3.8%–5.2%). A similarly significant effect was observed in patients with MBC (RD: 16.5%, 95% CI: 3.4%–29.6%; IPTW‐adjusted RD: 3.3%, 95% CI: 2.3%–4.4%).

In addition, the temporal trends in the proportions of NSM, Consis., and NST between 2010 and 2017 were evaluated and are presented in Figure 3b. Notably, the rates of NSM, Consis., and NST increased steadily during this period. The NSM rate increased from 1.9% in 2010 to 26.9% in 2017, the Consis. rate increased from 27.9% to 33.5%, and the NST rate increased from 20.5% to 34.9%.

### 3.5 Model Interpretation

SurvSHAP(t) is a new method that provides dynamic, time‐dependent explanations grounded in robust theoretical foundations. This approach was used to elucidate the outputs of the BIME model [32]. Aggregated Shapley values, computed across 500 observations, were utilized to identify and rank the eight most influential variables, as illustrated in Figure 3c. In this context, treatment‐related variables such as NSM and MRM in BIME represented transitions across distinct risk networks and used unique baseline hazards, distinguishing them from conventional covariates. The distribution of variable importance rankings—first, second, and subsequent positions—is illustrated by the horizontal bars, with different colors corresponding to each ranking tier.

## 4 Discussion

This study comprehensively evaluates the efficacy of various DL models, with a particular focus on the BIME model, which outperforms traditional manual treatment decisions, NCCN guidelines, and leading causal inference models. By rigorously mitigating baseline imbalances, the BIME model reduced mortality by 60% and extended survival by 18 months over a decade, highlighting its significant clinical potential.

The criteria for NSM in breast cancer treatment have expanded over time, but consensus on its application remains elusive [33]. The absence of RCTs directly comparing NSM and MRM complicates treatment effect extrapolation because of ethical and financial constraints. NSM is generally not recommended for advanced disease [34]. To address these challenges, this study applied two causal inference methods to balance prognostic factors between patient groups. The findings suggest that NSM is comparable or superior to MRM in OS and BCSS across all disease stages [35], likely reflecting advancements in diagnostic techniques that enhance NSM candidate selection [36] and innovations in surgical methods that reduce incision size, promoting faster recovery [37, 38]. NSM also significantly alleviates psychological burdens in patients with LABC, potentially improving long‐term compliance, satisfaction, and survival outcomes [39, 40]. However, baseline imbalances, such as a higher proportion of ER‐positive patients in the NSM group, may confer a prognostic advantage. Despite statistical adjustments, residual confounding may influence the observed ATE, necessitating cautious interpretation and further validation.

Although this study highlights the superiority of NSM over MRM in many aspects, other studies present differing perspectives. Some studies report significant benefits of NSM for early‐stage breast cancer, whereas its effectiveness may be lower than MRM in advanced cases [41, 42]. Furthermore, NSM indications for certain populations, particularly those with aggressive or advanced disease, remain contentious [43, 44], suggesting that surgical outcomes vary by patient cohort. Research consistently emphasizes the psychological benefits of NSM, including reduced postoperative stress and improved long‐term quality of life and surgical satisfaction [45, 46]. However, for advanced disease stages, some studies suggest that the psychological differences between NSM and MRM may be less pronounced [47], highlighting the heterogeneous mental health effects of surgical approaches.

For patients with EBC, our findings align with NCCN recommendations [48]. Among individuals with more advanced conditions, survival outcomes between NSM and MRM did not differ significantly, likely attributable to comparable surgical risks and the psychological impact of these procedures [38]. Nonetheless, additional studies are warranted to elucidate the mechanisms behind these observations.

NST has become a widely adopted approach for preserving breast tissue in breast cancer patients [49]. Recent analyses from the National Cancer Database indicate a growing trend favoring NSM following NST [11]. However, surgical decisions post‐NST are primarily guided by pathological response and physician experience [50], often lacking robust quantitative evidence. By using causal inference modeling, our study provides concrete evidence to inform surgical recommendations. Patients who underwent NST showed a 1.4% greater likelihood (95% CI: 0.9%–2.0%) of being recommended for NSM, with OS rates remaining consistent and key confounders addressed. This trend was particularly evident in patients with LABC, where the likelihood increased by 4.5% (95% CI: 3.8%–5.2%), consistent with data from a cohort study of 850 LABC patients [7]. Conversely, this effect was not statistically significant among patients with EBC, likely reflecting the already high prevalence of NSM adoption within this subgroup.

The development of a survival benefit visualization tool represents a critical advancement in supporting shared decision‐making between patients and physicians regarding therapeutic options. Such tools enable graphical comparisons of potential treatment outcomes by simulating counterfactual survival scenarios. While personalized treatment strategies and visual prognostic analyses are inherently complex [51, 52], BIME improves treatment accuracy through the integration of multiple causal inference methods. Accompanied by user‐friendly software, BIME demonstrates substantial potential for practical clinical application. However, several challenges must be addressed to facilitate its incorporation into routine clinical workflows. First, although rigorous statistical methods were applied in this study to mitigate confounding and selection bias, the possibility of residual unmeasured bias remains [53]. Future research should aim to validate the model using prospective study designs or emulate randomized clinical trials to bolster statistical robustness [54]. Second, clinical treatment decisions often extend beyond OS to encompass broader considerations, including quality of life and cost‐effectiveness. Incorporation of these additional outcome measures into BIME will empower patients to make more comprehensive and autonomous treatment decisions. Lastly, further refinement of the prognostic variables included in BIME, focusing on the selection of advanced and clinically relevant indicators, is essential to enhance the model's utility and applicability in diverse clinical contexts.

This study is subject to certain limitations. The lack of access to comprehensive data on treatment medications and dosages constrained the evaluation of the performance of our recommendations and the accuracy of the NST assessment. While OS serves as a central outcome measure, surgical decision‐making is influenced by various factors, including patient and physician preferences, socioeconomic status, and postoperative quality of life. These elements emphasize the necessity of incorporating a broader range of considerations into future research. Furthermore, validation of the proposed model using real‐world or prospective data is recommended to strengthen the applicability and reliability of the findings in clinical practice.

## 5 Conclusions

This study represents the first application of advanced deep learning models to inform surgical decision‐making in breast cancer patients, providing a quantitative evaluation of NST's role in determining surgical extent. The BIME model showed superior capability in identifying treatment heterogeneity, thereby facilitating evidence‐based clinical decision‐making. Notably, NST independently led to a 1.4% increase in surgical downgrading, with a particularly significant impact on surgical decisions for patients with LABC. This innovative approach underscores the transformative potential of DL in advancing precision medicine by tailoring treatment recommendations to the unique profiles of individual patients.

## Author Contributions

**Enzhao Zhu:** data curation (lead), formal analysis (lead), investigation (lead), methodology (lead), project administration (equal), resources (lead), software (lead), visualization (lead), writing – original draft, and writing – review and editing. **Linmei Zhang:** data curation (equal), formal analysis (equal), investigation (equal), methodology (equal), project administration (equal), visualization (equal), writing – original draft (equal), and writing – review and editing (equal). **Pu Ai:** conceptualization (equal), data curation (equal), methodology (equal), and writing – review and editing (equal). **Jiayi Wang:** methodology (equal) and writing – review and editing (equal). **Chunyu Hu:** methodology (equal) and software (equal). **Huiqing Pan:** investigation. **Weizhong Shi:** data curation and validation (equal). **Ziqin Xu:** methodology (equal) and resources (equal). **Yidan Fang:** investigation (equal), resources (equal). **Zisheng Ai:** funding acquisition (equal), supervision (equal), and validation (equal).

## Ethics Statement

The studies involving human participants were approved by the National Cancer Institution (approval ID: SAR0059979). The studies were conducted in accordance with the local legislation and institutional requirements.

## Consent

Written informed consent for participation was not needed for this study in accordance with national legislation and institutional requirements.

## Conflicts of Interest

The authors declare no conflicts of interest.

## Supporting information

Supporting information.

## Data Availability

This study utilized publicly available data from the Surveillance, Epidemiology, and End Results Program website (https://seer.gov/index.html).
