# Supplementary material for: Individualized Analysis of Nipple‐Sparing Mastectomy Versus Modified Radical Mastectomy Using Deep Learning
Source: Cancer Innov. 2025 Mar 26;4(3):e70002. doi: 10.1002/cai2.70002 (PMC11939007; doi:10.1002/cai2.70002)
Supplement: Supplementary file 1 — Supporting information. [file CAI2-4-e70002-s001.docx]

Supporting information

Table S1: Baseline demographic and pathological features following 1:1 propensity score matching

|  | Nipple-sparing mastectomy  (n=4,749) | Modified radical mastectomy  (n=33,410) | Statistics | *p* |
| --- | --- | --- | --- | --- |
| Age, mean (SD), y | 49.4 (10.6) | 49.4 (10.7) | −0.002^a^ | 0.997 |
| Axillary lymph node-positive, n (%) | 1,455 (30.7) | 1,455 (30.7) | 0^b^ | >0.999 |
| ER status–positive, n (%) | 3,952 (83.2) | 3,860 (81.3) | 5.971^b^ | 0.015* |
| PR status–positive, n (%) | 3,510 (73.9) | 3,511 (74.0) | 0^b^ | >0.999 |
| HER status-positive, n (%) | 873 (18.4) | 974 (18.4) | 0^b^ | >0.999 |
| Grade, n (%) |  |  | ...^c^ | >0.999 |
| I | 921 (19.4) | 920 (19.4) |  |  |
| II | 2,154 (45.4) | 2,154 (45.4) |  |  |
| III | 1,669 (35.2) | 1,670 (35.2) |  |  |
| IV | 3 (0) | 3 (0) |  |  |
| TNM stage, n (%) |  |  | 22.899^b^ | 0.002** |
| IA | 2,145 (45.2) | 2,145 (45.2) |  |  |
| IB | 130 (2.7) | 130 (2.7) |  |  |
| IIA | 1,274 (26.8) | 1,274 (26.8) |  |  |
| IIB | 701 (14.8) | 701 (14.8) |  |  |
| IIIA | 357 (7.5) | 357 (7.5) |  |  |
| IIIB | 34 (0.7) | 73 (1.5) |  |  |
| IIIC | 79 (1.7) | 49 (1.0) |  |  |
| IV | 29 (0.6) | 20 (0.4) |  |  |

a, Welch *t*-test; b, Chi-square test with continuity correction; c, Fisher’s exact test; *, *p* < 0.05; **, *p* < 0.01; ***, *p* < 0.001.

Figure S1: Standardized mean difference of IPTW and PSM correction


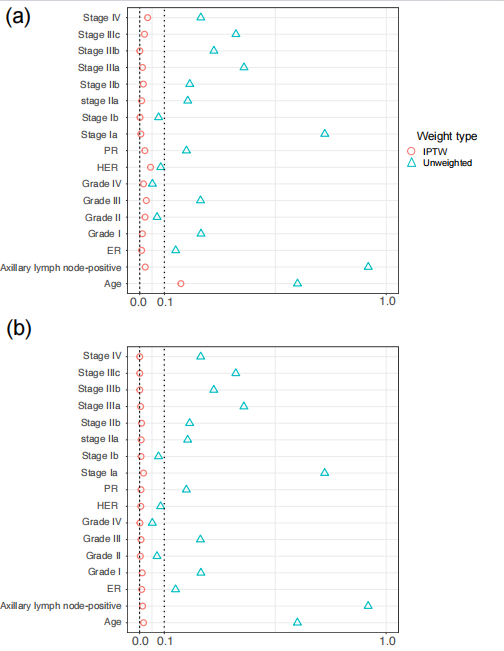


(a): Standardized mean difference for inverse probability treatment weighting. (b): Standardized mean difference for propensity score matching. IPTW, inverse probability treatment weighting; PSM, propensity score matching; HER, human epidermal growth factor receptor-2; PR, progesterone receptor; ER, estrogen receptor.

Figure S2: Causal path of model recommendation


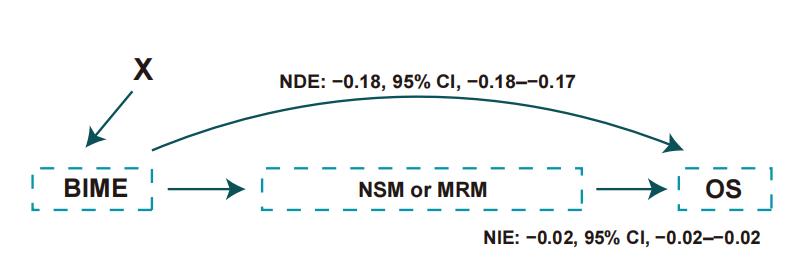


NDE, natural direct effect; NIE, natural indirect effect; BIME, Balanced Individual and Mixture Effect for survival regression; BCS, breast-conserving surgery; OS, overall survival; X, patient covariates.
